# Comparison of Injury Severity Score, Glasgow Coma Scale, and Revised Trauma Score in Predicting the Mortality and Prolonged ICU Stay of Traumatic Young Children: A Cross-Sectional Retrospective Study

**DOI:** 10.1155/2019/5453624

**Published:** 2019-12-01

**Authors:** Yii-Ting Huang, Ying-Hsien Huang, Ching-Hua Hsieh, Chao-Jui Li, I-Min Chiu

**Affiliations:** ^1^Department of Emergency Medicine, Kaohsiung Chang Gung Memorial Hospital, Kaohsiung, Taiwan; ^2^Department of Pediatrics, Chiayi Chang Gung Memorial Hospital, Puzi City, Taiwan; ^3^Department of Pediatrics, Kaohsiung Chang Gung Memorial Hospital and Chang Gung University College of Medicine, Kaohsiung, Taiwan; ^4^Department of Plastic Surgery, Kaohsiung Chang Gung Memorial Hospital, Chang Gung University and College of Medicine, Kaohsiung, Taiwan; ^5^Department of Computer Science and Engineering, National Sun Yat-Sen University, Kaohsiung, Taiwan

## Abstract

**Introduction:**

The purpose of this study was to examine the capacity of commonly used trauma scoring systems such as the Glasgow Coma Scale (GCS), Injury Severity Score (ISS), and Revised Trauma Score (RTS) to predict outcomes in young children with traumatic injuries.

**Methods:**

This retrospective study was conducted for the period from 2009 to 2016 in Kaohsiung Chang Gung Memorial Medical Hospital, a level I trauma center. We included all children under the age of 6 years admitted to the hospital via the emergency department with any traumatic injury and compared the trauma scores of GCS, ISS, and RTS on patients' outcome. The primary outcomes were mortality and prolonged Intensive Care Unit (ICU) stay, with the latter defined as an ICU stay longer than 14 days. The secondary outcome was the hospital length of stay (HLOS). Receiver operating characteristic (ROC) analysis was also adopted with the value of the area under the ROC curve (AUC) for comparing trauma score prediction with patient mortality. Cutoff values from each trauma score for mortality prediction were also measured by determining the point along the ROC curve where Youden's index was maximum.

**Results:**

We included a total of 938 patients in this study, with a mean age of 3.1 ± 1.82 years. The mortality rate was 0.9%, and 93 (9.9%) patients had a prolonged ICU stay. An elevated ISS (34 ± 19.9 vs. 5 ± 5.1, *p*=0.004), lower GCS (8 ± 5.0 vs. 15 ± 1.3, *p*=0.006), and lower RTS (5.58 ± 1.498 vs. 7.64 ± 0.640, *p*=0.006) were all associated with mortality. All three scores were considered to be independent risk factors of mortality and prolonged ICU stay and had a linear correlation with increased HLOS. With regard to predicting mortality, ISS has the highest AUC value (ISS: 0.975; GCS: 0.864; and RTS: 0.899). The prediction cutoff values of ISS, GCS, and RTS on mortality were 15, 11, and 7, respectively.

**Conclusion:**

Regarding traumatic injuries in young children, worse ISS, GCS, and RTS were all associated with increased mortality, prolonged ICU stay, and longer hospital LOS. Of these scoring systems, ISS was the best at predicting mortality.

## 1. Introduction

Trauma is considered a big threat to childhood survival. The National Center for Health Statistics in the United States has indicated that unintentional injury is the leading cause of death and disability in children [1]. Managing pediatric trauma events at the emergency department (ED) is often a challenge because children have less fat and more elastic connective tissue covering a flexible skeleton that protects packed abdominal and thoracic structures [2]. As a result, the impact and causes of trauma can vary considerably among different age groups. The anatomical, physiological, and emotional differences between adults and children imply that children are not just adults on a smaller scale [3].

In the past, most studies related to trauma assessment have investigated pediatric patients with ages ranging from about 0–18 years [4, 5]. Nevertheless, age-based studies focused on younger victims have not been common. Managing traumatic injury in young children is different from adults as children's compensatory responses to large numbers of blood loss, hypoxia, severe trauma, and burns differ significantly [6]. Furthermore, young patients often do not have enough vocabulary, resulting in limited expression, especially for children under two years of age [7, 8]. Young children also have difficulty in accurately expressing their feelings when a medical history is taken and are often irritated and crying upon arriving at the hospital due to pain or fright. Therefore, trauma scores related to vital signs and physical examinations are more objective and accessible [9]. Obtaining trauma scores earlier allows critical intervention. In this study, we aimed to investigate several commonly used traumatic scores on the outcomes of young children, including mortality, prolonged ICU stay, and hospital length of stay (HLOS).

## 2. Materials and Methods

### 2.1. Trauma Scores Selection

Various quantitative scoring systems have been proposed to evaluate trauma severity and outcome, but most of them were not age specific, and each had its own limitations [10–13]. Considering the different physiological structures in younger children, we selected the Injury Severity Score (ISS), which emphasizes anatomic criteria and has been validated to predict prognosis [14]. In previous studies, major trauma in the pediatric category has been defined as an Injury Severity Score greater than 15 [15, 16]. However, few studies have focused on ISS performance in young children [17]. Despite a number of proposed modifications and alternate scoring systems, ISS remains the most widely used to define severely injured patients, which is why we chose it [16, 18, 19].

The Glasgow Coma Scale (GCS) indicates level of consciousness and has always been evaluated upon patient arrival. This scale has been frequently used for decades as blunt head trauma is a common cause of mortality and morbidity in pediatric injuries [20–23]. Since head injury is one of the most common traumatic mechanisms in young children, GCS is also appropriate for our study's main group.

The Revised Trauma Score (RTS) is used in prehospital practices worldwide, can be obtained immediately, and includes the GCS, systolic blood pressure, and respiratory rate. The formula for calculating RTS is as follows: RTS=0.7326 *∗* systolic blood pressure+0.2908 *∗* respiratory rate+0.9368 *∗* GCS [24].

### 2.2. Study Population and Design

This retrospective study was conducted from January 1, 2009, to December 31, 2016, in Kaohsiung Chang Gung Memorial Medical Hospital, a level I trauma center in southern Taiwan. The Institutional Review Board of the Chang Gung Medical Foundation approved the medical information, and all patients' and physicians' records and information were anonymized and deidentified prior to analysis (IRB number: 201801721B0). Additional detailed information of all trauma patients was retrieved from the studied institution's trauma registry system, including age, gender, initial vital signs, cause of injury, different types of trauma severity scores, hospital length of stay (LOS), intensive care unit (ICU) stay, and mortality.

This study consisted of children under the age of 6 years with any type of traumatic injury who were admitted to the hospital via the ED. Patients with a pre-existing medical condition that contributed to the trauma incident and who died in the ED were excluded. We used vital signs and GCSs at triage for scoring, comparing the accuracy of the GCS, ISS, and RTS trauma scores on predicting patients' outcome. Initial triage and vital signs were obtained by senior and well-trained emergency nurses, and child-version sphygmomanometer was used for young children. ISS was measured by the trauma physicians in charge in the ED.

The primary outcomes were trauma-related mortality during admission and prolonged ICU stay, which was defined as an ICU stay longer than 14 days. Prolonged ICU stay is usually defined as ≥14 days admission in the ICU, which has been considered with resource utilization and patients' morbidity and mortality [25–29]. The HLOS was considered as the secondary outcome in patients who survived beyond admission. Patients who expired before admission were excluded from prolonged ICU stay and HLOS analysis.

### 2.3. Statistical Analysis

Trauma scores' contribution to outcomes including mortality and prolonged ICU stay was analyzed by Student's *t*-test and validated using binary regression after adjusting for age, gender, and cause of injury. Linear regression was used to observe the correlation between trauma scores and HLOS.

We further drew the receiver operating characteristic (ROC) curve and calculated the value of the area under the ROC curve (AUC) to compare trauma scores prediction with patient mortality. Cutoff values from each trauma score for mortality prediction were also measured by determining the point along the ROC curve where Youden's index was maximum [30]. The capability of trauma scores in predicting the mortality and prolonged ICU stay was calculated using the Chi-squared test. All statistical analysis was conducted with SPSS (version 22).

## 3. Results

This study consisted of a total of 938 patients, with a mean age of 3.1 ± 1.82 years. The overall mortality rate was 0.9%. The rate of ICU stay was 41.7% (*N*=391), with a mean ICU stay of 10.2 ± 7.83 days. Regarding the cause of trauma, injury from fall, traffic accident, and burn injury accounted for 86.7% of all admitting injuries. The average trauma scores of ISS, GCS, and RTS in the general population were 4.9 ± 6.0, 14.7 ± 1.4, and 7.62 ± 0.68, respectively (Table 1). We excluded 25 patients from RTS and outcome analysis since their initial blood pressure had not been obtained.

Table 2 shows the trauma scores between patients of both the mortality and survival groups compared with student's *t*-test. The ISS score was statistically higher in the mortality group (34 ± 19.9 vs. 5 ± 5.1, *p*=0.004), while GCS (8 ± 5.0 vs. 15 ± 1.3, *p*=0.006) and RTS (5.58 ± 1.498 vs. 7.64 ± 0.640, *p*=0.006) scores were lower. When comparing different trauma scores with prolonged ICU stay, only RTS demonstrated a significant difference between the two studied groups (7.34 ± 1.019 vs. 7.67 ± 0.577; *p*=0.004) (Table 2).

As shown in Table 3, the logistic regression regarding trauma scores' in association with primary and secondary outcomes is displayed. ISS (aOR: 1.17, 95% CI: 1.091–1.225), GCS (aOR: 0.59, 95% CI: 0.492–0.714), and RTS (aOR: 0.19, 95% CI: 0.094–0.373) were all considered to be independent risk factors of mortality and prolonged ICU stay. In addition, younger age and burn injury were also risk factors for prolonged ICU stay. All three trauma scores' changes had a positive effect on HLOS, and these effects were found to be statistically significant (Table 3).

**Figure 1:**
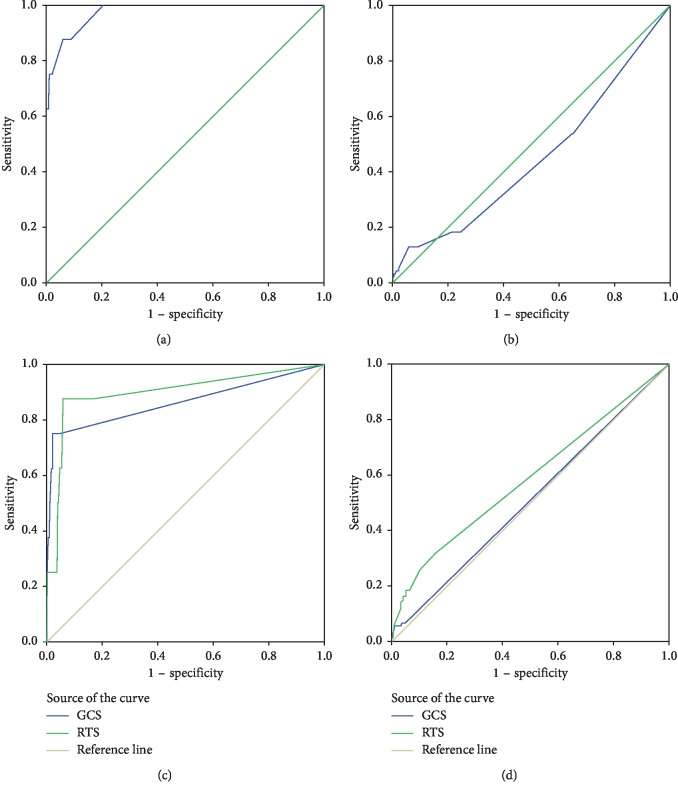
ROC curve of trauma scores to mortality and prolonged ICU stay. (a) ISS to mortality. (b) ISS to prolonged ICU stay. (c) GCS and RTS to mortality. (d) GCS and RTS to prolonged ICU stay.

Figure 1 shows the ROC curves of different trauma scores related to outcomes. Upon calculating the AUC value (Table 4), all trauma scores revealed acceptable prediction ability for mortality (ISS: 0.975, 95% CI: 0.940∼1; GCS: 0.864, 95% CI: 0.682∼1; RTS: 0.899, 95% CI: 0.759∼1) but not for prolonged ICU stay (ISS: 0.502, 95% CI: 0.384∼0.513; GCS: 0.426, 95% CI: 0.447∼0.572; RTS: 0.578, 95% CI: 0.520∼0.653). We further measured the cutoff value for mortality prediction of each trauma score according to the ROC curves, which were found to be 15 for ISS, 11 for GCS, and 7 for RTS.

The evaluation of each trauma score's cutoff value regarding mortality is provided in Table 5. Using ISS ≥15 as the cutoff value for predicting mortality, its positive predictive value (PPV) was 11.1%, while its negative predictive value (NPV) was 99.9% (*p* < 0.001, OR = 109.25). Meanwhile, GCS ≤11 had a PPV of 23.1% and NPV of 99.8% for predicting mortality (*p* < 0.001, OR = 136.50). Finally, using RTS ≤7 as a predictor for mortality resulted with a PPV of 9.9% and NPV of 99.9% (*p* < 0.001, OR = 94.72).

## 4. Discussion

In this study, we included all patients admitted to the trauma center younger than the age of 6 years, with a mean age of 3.1 years and an overall mortality rate of 0.9%. As in previous studies, the mortality rate of pediatric trauma was approximately 2% [31, 32]. The three most common causes of trauma in this study were, in order, injury from fall, traffic accident, and burn injury. Adegoke et al. reported similar findings, while Derakhshanfar et al. reported traffic accidents to be the most common cause, followed by fall injuries [33–35].

The trauma score systems selected in this study were ISS, GCS, and RTS since their values were easy to obtain and calculate. Furthermore, consciousness level is the main domain of these trauma scores since brain injury is the primary cause of mortality and morbidity in pediatric injuries [36]. The average trauma scores of ISS, GCS, and RTS in our studied population were 4.9, 14.7, and 7.62, respectively. In contrast, the average trauma scores were 34, 8, and 5.58 among patients who died. In previous studies, these trauma scores in survivor and mortality populations have traditionally varied. Yousefzadeh Chabok et al. demonstrated an ISS of 6.5 overall and 17.7 in the mortality group, with GCS scores of 4.7 in the mortality group and 14.6 in the survivor group [32]. Soni et al. showed RTS scores of 7.13 in trauma survivors and 4.39 in nonsurvivors, with ISS scores of 11.68 in the mortality group and 11.87 in the survivor group [37]. In our study, all trauma scores differed with statistical significance between the survivor and mortality groups.

After adjusting for gender, age, and traumatic mechanism using binary regression, lower GCS and RTS levels still appeared to be associated with increased mortality and prolonged ICU stay. Increased ISS scores also have a positive effect on higher mortality and prolonged ICU stay rates, though the latter did not demonstrate statistical difference.

As for secondary outcomes, all three trauma scores demonstrated a linear correlation with hospital length of stay after adjusting for age, gender, and trauma mechanism (Table 4). This result was similar to a previous study in China that showed that increased ISS and decreased RTS were correlated with an increased length of hospital stay [38].

Regarding mortality prediction, ROC curve analysis indicated that all three scoring systems were statistically significant (Table 4). While some previous studies demonstrated that GCS can better predict mortality [32, 39], in our study, although the difference was small, we showed ISS to be the strongest predictor of mortality. However, none of the three trauma scoring systems could predict prolonged ICU stays with significance.

The cutoff values selected from the ROC analysis of ISS, GCS, and RTS were 15, 11, and 7, respectively. A similar cutoff value for ISS was reported in a previous study by Yousefzadeh Chabok et al. [38]. Both the sensitivity and specificity for mortality prediction regarding the selected cutoff values from each trauma score system were high (Table 5). However, all the selected cutoff values had much higher NPV than PPV on mortality prediction, which suggests that the cutoff values of all three trauma score systems were better at predicting survival than mortality.

## 5. Limitations

This study has certain limitations. This retrospective study was conducted in a single medical center, which may limit the generalizability of the conclusions. All possible confounding factors were unmodifiable, and a cause-and-effect relationship could not be determined. The assessment of trauma scores was performed by different trained medical personnel throughout the study period, which may have led to interpersonal bias.

Furthermore, the overall mortality rate of our study was 0.9% among the 938 patients, which was less than our reference in previous literature, and the accuracy in using trauma scoring systems may differ. Moreover, since the most traumatic mechanism in our study was burn injuries, an ISS may be falsely elevated when a patient has minor burns on various body parts. In contrast, the severity of burn injuries may be underestimated by trauma scoring systems in a patient with severe, widespread cutaneous burns.

Despite these limitations, the results from this study can help distinguish low-risk patients for mortality and prolonged ICU stay from high-risk ones and may improve ED disposition in clinical practice. Very few studies have investigated trauma scoring and prognosis for young children under the age of 7 years. Further research on the association of trauma scores with prognosis in specific types of accidents is needed in the future.

## 6. Conclusion

In this study, we found that worse trauma scores of ISS, GCS, and RTS were associated with increased mortality, prolonged ICU stays, and HLOS among young children's injuries. Among these three trauma scores, we found ISS to have the best predictive value. The cutoff values of ISS, GCS, and RTS for predicting mortality were 15, 11, and 7, respectively.

## Figures and Tables

**Table 1:** Epidemiology of trauma patients under the age of six years admitted via the pediatric emergency department.

	Variables	Number (%)/mean ± SD
	Total	938 (100%)
	Age (years)	3.1 ± 1.82
	BMI	16.6 ± 4.40
Gender	Male	554 (59.1%)
Cause of injury	Injury from fall	329 (35.1%)
	Traffic accident	134 (14.3%)
	Burn injury	351 (37.4%)
	Blunt/crushing injury	71 (7.6%)
	Other	53 (5.7%)
Outcome	Mortality	8 (0.9%)
	ICU admission	391 (41.7%)
	Prolonged ICU stay	93 (9.9%)
	Length of ICU stay (days)	10.2 ± 7.83
	Length of hospital stay (days)	6.9 ± 7.69
Trauma score	ISS	4.9 ± 6.0
	GCS	14.7 ± 1.4
	RTS	7.62 ± 0.68

BMI = body mass index; ICU = intensive care unit; ISS = Injury Severity Score; GCS = Glasgow Coma Scale; RTS = Revised Trauma Score.

**Table 2:** Comparison of trauma scores in different groups of mortality and prolonged ICU stay.

Trauma score	Mortality	Survival	*p*-value	ICU stay ≥ 14 days	ICU stay < 14 days	*p*-value
	Mean ± SD			Mean ± SD		
ISS	34 ± 19.9	5 ± 5.1	**0.004**	4 ± 5.0	5 ± 5.1	0.237
GCS	8 ± 5.0	15 ± 1.3	**0.006**	15 ± 1.8	15 ± 1.2	0.415
RTS	5.58 ± 1.498	7.64 ± 0.640	**0.006**	7.34 ± 1.019	7.67 ± 0.577	**0.004**

SD = standard deviation; ISS = Injury Severity Score; GCS = Glasgow Coma Scale; RTS = Revised Trauma Score.

**Table 3:** Logistic regression for trauma score to mortality, prolonged ICU stay, and hospital length of stay (after adjusting for age, gender, and cause of injury).

Variables	aOR	95% CI	aOR	95% CI	*β* coefficient	95% CI
	*Mortality*		*Prolonged ICU stay*		*Hospital length of stay*	
Age	0.983	0.609–1.625	0.809^*∗*^	0.707–0.925	−0.602^*∗*^	−0.849∼−0.355
Male	3.293	0.353–30.594	1.021	0.651–1.602	−0.426	−1.311∼0.459
Injury from fall	1.239	0.188–8.148	0.079^*∗*^	0.029–0.218	−6.099^*∗*^	−7.016∼−5.183
Traffic accident	3.393	0.453–25.155	0.122^*∗*^	0.029–0.516	−1.511^*∗*^	−2.911∼−0.111
Burn injury	0.092	0.001–14.410	18.199^*∗*^	8.931–37.085	8.609^*∗*^	7.701∼9.516
Blunt/crushing injury	—	—	0.526	0.186–1.491	−1.317	−3.049∼0.414
Others	—	—	0.532	0.161–1.752	−1.032	−3.015∼0.952
**ISS**	**1.17**	**1.091**–**1.225**	**1.034**	**1.004**–**1.063**	**0.315**	**0.224**∼**0.401**
Age	1.164	0.726–1.865	0.818	0.715–0.936	−0.536^*∗*^	−0.792∼−0.280
Male	1.053	0.194–5.727	0.99	0.631–1.552	−0.437	−1.353∼0.479
Injury from fall	0.550	0.084–3.614	0.081^*∗*^	0.029–0.225	−5.497^*∗*^	−6.434∼−4.559
Traffic accident	3.788	0.719–19.949	0.005^*∗*^	0.029–0.525	−0.505	−1.890∼0.879
Burn injury	1.062	0.142–7.939	15.92^*∗*^	8.046–31.500	7.198^*∗*^	6.625∼8.130
Blunt/crushing injury	0	—	0.497	0.174–1.420	−1.682	−3.438∼0.074
Others	0	—	0.526	0.160–1.733	−1.441	−3.451∼0.569
**GCS**	**0.59**	**0.492**–**0.714**	**0.82**	**0.692**–**0.970**	−**0.783**	−**1.174**∼−**0.393**
Age	1.334	0.857–2.076	0.853	0.744–0.977	−0.406	−0.663∼−0.149
Male	1.134	0.225–5.719	1.041	0.661–1.639	−0.317	−1.222∼0.589
Injury from fall	1.159	0.194–6.922	0.085^*∗*^	0.031–0.234	−5.287	−6.217∼4.356
Traffic accident	8.822^*∗*^	1.662–46.835	0.131^*∗*^	0.031–0.547	−0.357	−1.713∼0.999
Burn injury	0.143	0.019–1.078	12.591^*∗*^	6.675–23.749	6.703	5.775∼7.631
Blunt/crushing injury	0	—	0.531	0.186–1.518	−1.463	−3.196∼0.270
Others	0	—	0.548	0.166–1.812	−1.311	−3.294∼−0.673
**RTS**	**0.19**	**0.094**–**0.373**	**0.69**	**0.534**–**0.896**	−**2.981**	−**4.209**∼−**1.752**

**Table 4:** AUC value of trauma score to mortality and prolonged ICU stay.

	Mortality			Prolonged ICU stay		
	AUC	Std. error	95% CI	AUC	Std. error	95% CI
ISS	0.975	0.018	0.940∼1	0.448	0.033	0.384∼0.513
GCS	0.864	0.093	0.682∼1	0.509	0.032	0.447∼0.572
RTS	0.899	0.071	0.759∼1	0.587	0.034	0.520∼0.653

**Table 5:** Association of trauma score's cutoff value with mortality.

		Number	Mortality rate *N* (%)	*p*-value	Odds ratio
ISS	ISS ≥ 15	63	7 (11.1%)	<0.001	109.25
	ISS < 15	875	1 (0.1%)		
GCS	GCS ≤ 11	26	6 (23.1%)	<0.001	136.50
	GCS > 11	912	2 (0.2%)		
RTS	RTS ≤ 7	71	7 (9.9%)	<0.001	94.72
	RTS > 7	867	1 (0.1%)		

## Data Availability

Raw data were generated at Chang Gung Memorial Medical Hospital. Derived data supporting the findings of this study are available from the corresponding author on request.

## References

[1] National Center for Injury Prevention and Control CuW (2017). Leading causes of death reports. 1981–2016. https://webappa.cdc.gov/sasweb/ncipc/leadcause.html.

[2] Stafford P. W., Blinman T. A., Nance M. L. (2002). Practical points in evaluation and resuscitation of the injured child. *Surgical Clinics of North America*.

[3] McFadyen J., Ramaiah R., Bhananker S. (2012). Initial assessment and management of pediatric trauma patients. *International Journal of Critical Illness and Injury Science*.

[4] Muisyo T., Bernardo E. O., Camazine M. (2019). Mortality prediction in pediatric trauma. *Journal of Pediatric Surgery*.

[5] McCarty T. R., Abramo T. J., Maxson R. T. (2018). Hypothermia as an outcome predictor tool in pediatric trauma: a propensity-matched analysis. *Pediatric Emergency Care*.

[6] Loomis A. (2018). The role of preschool as a point of intervention and prevention for trauma-exposed children: recommendations for practice, policy, and research. *Topics in Early Childhood Special Education*.

[7] El-Sheikh M., Cheskes J. (1995). Background verbal and physical anger: a comparison of children’s responses to adult-adult and adult-child arguments. *Child Development*.

[8] Bernstein Ratner N. (1992). Measurable outcomes of instructions to modify normal parent-child verbal interactions: implications for indirect stuttering therapy. *Journal of Speech, Language, and Hearing Research*.

[9] Reilly P. L., Simpson D. A., Sprod R., Thomas L. (1988). Assessing the conscious level in infants and young children: a paediatric version of the Glasgow coma scale. *Child’s Nervous Systems*.

[10] Timothy H., Pohlman M. (May 2014). FACS. Trauma scoring systems. https://emedicine.medscape.com/article/434076-overview.

[11] Chawda M. N., Hildebrand F., Pape H. C., Giannoudis P. V. (2004). Predicting outcome after multiple trauma: which scoring system?. *Injury*.

[12] Köksal O., Ozdemir F., Bulut M., Aydin S., Almacioğlu M. L., Ozgüç H. (2009). Comparison of trauma scoring systems for predicting mortality in firearm injuries. *Ulusal Travma ve Acil Cerrahi Dergisi*.

[13] Marcin J. P., Pollack M. M. (2002). Triage scoring systems, severity of illness measures, and mortality prediction models in pediatric trauma. *Critical Care Medicine*.

[14] Walker P. J., Cass D. T. (1987). Paediatric trauma: urban epidemiology and an analysis of methods for assessing the severity of trauma in 598 injured children. *ANZ Journal of Surgery*.

[15] Copes W. S., Champion H. R., Sacco W. J., Lawnick M. M., Keast S. L., Bain L. W. (1988). The injury severity score revisited. *The Journal of Trauma: Injury, Infection, and Critical Care*.

[16] Palmer C. (2007). Major trauma and the injury severity score–where should we set the bar?. *Annual Proceedings. Association for the Advancement of Automotive Medicine*.

[17] Brown J. B., Gestring M. L., Leeper C. M. (2017). The value of the injury severity score in pediatric trauma. *Journal of Trauma and Acute Care Surgery*.

[18] Abbasi H. R., Mousavi S. M., Taheri Akerdi A., Niakan M. H., Bolandparvaz S., Paydar S. (2013). Pattern of traumatic injuries and injury severity score in a major trauma center in Shiraz, Southern Iran. *Bulletin of Emergency and Trauma*.

[19] Maduz R., Kugelmeier P., Meili S., Döring R., Meier C., Wahl P. (2017). Major influence of interobserver reliability on polytrauma identification with the injury severity score (ISS): time for a centralised coding in trauma registries?. *Injury*.

[20] Holmes J. F., Palchak M. J., MacFarlane T., Kuppermann N. (2005). Performance of the pediatric Glasgow coma scale in children with blunt head trauma. *Academic Emergency Medicine*.

[21] Mayer T., Walker M. L., Johnson D. G., Matlak M. E. (1981). Causes of morbidity and mortality in severe pediatric trauma. *JAMA: The Journal of the American Medical Association*.

[22] Tepas J. J., DiScala C., Ramenofsky M. L., Barlow B. (1990). Mortality and head injury: the pediatric perspective. *Journal of Pediatric Surgery*.

[23] Teasdale G. M., Pettigrew L. E. L., Wilson J. T. L., Murray G., Jennett B. (1998). Analyzing outcome of treatment of severe head injury: a review and update on advancing the use of the Glasgow outcome scale. *Journal of Neurotrauma*.

[24] Gilpin D. A., Nelson P. G. (1991). Revised trauma score: a triage tool in the accident and emergency department. *Injury*.

[25] Tobi K. U., Amadasun F. E. (2015). Prolonged stay in the intensive care unit of a tertiary hospital in Nigeria: predisposing factors and outcome. *African Journal of Medical and Health Sciences.*.

[26] Arabi Y., Venkatesh S., Haddad S., Al Shimemeri A., Al Malik S. (2002). A prospective study of prolonged stay in the intensive care unit: predictors and impact on resource utilization. *International Journal for Quality in Health Care*.

[27] Zampieri F. G., Ladeira J. P., Park M. (2014). Admission factors associated with prolonged (>14 days) intensive care unit stay. *Journal of Critical Care*.

[28] Laupland K. B., Kirkpatrick A. W., Kortbeek J. B., Zuege D. J. (2006). Long-term mortality outcome associated with prolonged admission to the ICU. *Chest*.

[29] Chalya P. L., Gilyoma J. M., Dass R. M. (2011). Trauma admissions to the intensive care unit at a reference hospital in Northwestern Tanzania. *Scandinavian Journal of Trauma, Resuscitation and Emergency Medicine*.

[30] Habibzadeh F., Habibzadeh P., Yadollahie M. (2016). On determining the most appropriate test cut-off value: the case of tests with continuous results. *Biochemia Medica*.

[31] Brysiewicz P., Clarke D. L., Sartorius B., Bruce J. L., Laing G. L. (2017). Defining predictors of mortality in pediatric trauma patients. *South African Journal of Surgery*.

[32] Yousefzadeh-Chabok S., Kazemnejad-Leili E., Kouchakinejad-Eramsadati L. (2016). Comparing pediatric trauma, Glasgow coma scale and injury severity scores for mortality prediction in traumatic children. *Ulusal Travma ve Acil Cerrahi Dergisi*.

[33] Franzén L., Örtenwall P., Backteman T. (2007). Children in Sweden admitted to intensive care after trauma. *Injury*.

[34] Adegoke S. A., Oginni L. M. (2011). Predictors of paediatric injury mortality. *South Africa Journal of Child Health*.

[35] Derakhshanfar H., Hatamabadi H., Karimian K. (2012). The prognosis of trauma among children and the factors contributing to it. *Health*.

[36] Morrissey K., Fairbrother H., Vazquez M. N. (2016). Severe traumatic brain injury in children: an evidence-based review of emergency department management [digest]. *Pediatric Emergency Medicine Practice*.

[37] Soni K. D., Mahindrakar S., Gupta A., Kumar S., Sagar S., Jhakal A. (2017). Comparison of ISS, NISS, and RTS score as predictor of mortality in pediatric fall. *Burns & Trauma*.

[38] Yousefzadeh Chabok S., Ranjbar Taklimie F., Malekpouri R., Razzaghi A. (2017). Predicting mortality, hospital length of stay and need for surgery in pediatric trauma patients. *Chinese Journal of Traumatology*.

[39] Cantais E., Paut O., Giorgi R., Viard L., Camboulives J. (2001). Evaluating the prognosis of multiple, severely traumatized children in the intensive care unit. *Intensive Care Medicine*.

